# Intravenous ferric derisomaltose versus oral iron for persistent iron deficient pregnant women: a randomised controlled trial

**DOI:** 10.1007/s00404-022-06768-x

**Published:** 2022-09-15

**Authors:** Rebecka Hansen, Veronika Markova Sommer, Anja Pinborg, Lone Krebs, Lars Lykke Thomsen, Torben Moos, Charlotte Holm

**Affiliations:** 1 Department of Obstetrics and Gynaecology, Copenhagen University Hospital Hvidovre, Hvidovre, Denmark; 2 Fertility Department, Copenhagen University Hospital Rigshospitalet, Copenhagen, Denmark; 3 Department of Clinical Medicine, University of Copenhagen, Copenhagen, Denmark; 4 Department of Clinical and Non-Clinical Research, Pharmacosmos A/S, Holbæk, Denmark; 5 Neurobiology and Drug Delivery, Department of Health Science and Technology, Aalborg University, Aalborg, Denmark

**Keywords:** Iron deficiency, Anaemia, Pregnancy, Intravenous iron

## Abstract

**Purpose:**

To compare the efficacy of intravenous (IV) iron (ferric derisomaltose) with oral iron (ferrous fumarate) in women 14–21 weeks pregnant with persistent iron deficiency (ferritin < 30 µg/L).

**Methods:**

In a single-centre, open-label, randomised controlled trial at a Danish hospital, women with persistent iron deficiency after routine oral iron treatment were allocated to receive 1000 mg IV iron (single-dose) or 100 mg elemental oral iron daily. Outcomes were assessed during an 18-week follow-up period. The primary endpoint was the proportion of non-anaemic (haemoglobin [Hb] ≥ 11 g/dL) women throughout follow-up. Other outcomes included changes in haematological parameters, patient-reported fatigue, and quality of life (QoL). Safety was assessed by recording adverse events.

**Results:**

From July 2017 to February 2020, 100 women were randomised to IV iron and 101 to oral iron. Throughout follow-up, 91% of women were non-anaemic in the IV iron group compared with 73% in the oral iron group (18% difference [95% confidence interval 0.10–0.25]; *p* < 0.001). The mean Hb increase was significantly greater with IV iron versus oral iron at Weeks 6 (0.4 versus − 0.2 g/dL; *p* < 0.001), 12 (0.5 versus 0.1 g/dL; *p* < 0.001), and 18 (0.8 versus 0.5 g/dL; *p* = 0.01). Improvements in fatigue and QoL were greater with IV iron versus oral iron at Weeks 3 and 6. The incidence of treatment-related adverse events was comparable between treatment groups.

**Conclusion:**

IV iron was superior in preventing anaemia compared with oral iron in pregnant women with persistent iron deficiency; biochemical superiority was accompanied by improved fatigue and QoL.

**Clinical trial registration:**

European Clinical Trials Database: EudraCT no.: 2017-000776-29 (3 May 2017); ClinicalTrials.gov: NCT03188445 (13 June 2017).

The trial protocol has been published: https://dx.doi.org/10.1186%2Fs13063-020-04637-z.

**Supplementary Information:**

The online version contains supplementary material available at 10.1007/s00404-022-06768-x.

## What does this study add to the clinical work

**Table:** 

Intravenous iron is an effective and safe alternative to oral iron for the treatment of persistent iron deficiency in pregnant women	

## Introduction

Worldwide, about 38% of pregnant women are anaemic (haemoglobin [Hb] < 11.0 g/dL) [1]. In Europe, approximately one in four pregnant women have anaemia, and roughly half of these cases are due to iron deficiency (ID) [1]. ID has been estimated to affect around 40% of all pregnant women [2] and is highly prevalent in Europe [3].

During pregnancy, iron requirements increase due to placental growth, foetal development and enhanced erythropoiesis [4]. If iron intake is low, stored body iron can become depleted, ultimately compromising erythropoiesis, and causing iron deficiency anaemia (IDA) [5]. IDA has been associated with a reduced maternal physical and intellectual capacity [6] and low birth weight [7–9]. Non-anaemic ID has also emerged as a potential risk for low birth weight [8] and compromised foetal neurodevelopment with long-term consequences [10, 11].

Oral iron is frequently used in the routine treatment of ID and IDA in pregnant women. It is inexpensive, but often poorly tolerated, which can compromise compliance [12]. An alternative to oral iron treatment is intravenous (IV) iron, which has been associated with improved Hb and iron status at term in pregnant women initially diagnosed with anaemia [13–16]. This trial aims to compare the efficacy and safety of IV iron with oral iron for the prevention of anaemia in pregnant women who have persistent ID despite routine oral iron treatment.

## Methods

### Study design, setting and population

A single-centre, open-label, randomised, comparative trial was conducted between July 2017 and February 2020 at the Department of Obstetrics and Gynaecology, Copenhagen University Hospital Hvidovre, Denmark. The department employed routine screening for ID and anaemia in the first trimester by measuring Hb and ferritin levels. ID was defined as a ferritin level < 30 µg/L—a threshold with good diagnostic accuracy for determining low storage iron [17], which is commonly used in clinical trials [5], and in practice, to guide therapy [5, 18]. Women with ID were recommended oral iron doses of 60–100 mg daily, depending on the severity of ID. Women with persistent ID, (ferritin < 30 µg/L after approximately four weeks of treatment with oral iron) were invited to participate in the trial.

Participants were randomly assigned to receive either a single dose of IV iron (ferric derisomaltose [FDI], also known as iron isomaltoside; Monofer^®^/Monoferric^®^, Pharmacosmos A/S, Holbæk, Denmark) or to continue daily treatment with oral iron (ferrous fumarate and ascorbic acid; Jern C^®^, Mylan Denmark Aps, Ballerup, Denmark), using a 1:1 allocation ratio stratified by Hb level (Hb ≥ or < 11.0 g/dL at the last measurement prior to inclusion). Random assignment was performed at the baseline visit using the IBM Clinical Development interactive web response system and cloud-based electronic data capture platform. Recruitment, randomisation, drug administration, and data collection were carried out by the investigators.

Key inclusion criteria included: women aged ≥ 18 years; pregnant in the second trimester (gestational age 14–21 weeks, inclusive); persistent ID (ferritin < 30 µg/L) after four weeks of treatment with oral iron. Exclusion criteria included multiple pregnancies, a history of multiple allergies, known hypersensitivity to any of the excipients in the investigational drugs, active infections, and recent red blood cell (RBC) transfusion. All randomised participants had four scheduled follow-up visits at 3, 6, 12 and 18 weeks post baseline.

### Treatments

Treatment was initiated at the baseline visit. After baseline data were collected, women allocated to the IV iron group received a single dose of 1000 mg FDI diluted in 100 mL 0.9% sodium chloride (or if pre-pregnancy body weight was < 50 kg, the dose was 20 mg/kg based on pre-pregnancy body weight). Infusions were administered over approximately 20 min and participants were observed for adverse reactions during, and 30 min after, the end of the infusion. Women in the oral iron group received ferrous fumarate tablets (equivalent to 100 mg elemental iron) combined with 60 mg ascorbic acid for self-administration once daily throughout the trial (i.e., 18 weeks). Compliance was encouraged at each visit and assessed by pill count at Weeks 6 and 18. Regardless of initial allocation, all participants presenting with IDA (Hb < 11.0 g/dL and ferritin < 30 µg/L) at the 6 or 12-week visits were offered an additional IV iron infusion, dosed and administered in the same way as described for the baseline visit. In women who received additional IV iron at, or after, 26 weeks gestational age, foetal heart rate was monitored with cardiotocography. Participants in the oral iron group who received additional IV iron stopped oral iron therapy when IV treatment was initiated.

### Efficacy assessment

Efficacy outcomes were assessed through blood sampling and questionnaire completion, which occurred at all trial visits. The primary efficacy endpoint assessed the avoidance of anaemia by determining the proportion of women with Hb ≥ 11.0 g/dL (≥ 6.8 mmol/L) at all follow-up visits during the 18-week trial period. Secondary efficacy outcomes included changes in haematological parameters (Hb, ferritin, and transferrin saturation [TSAT]) and changes in patient-reported outcomes (PROs). PROs included fatigue and quality of life (QoL), which were assessed at each visit by the official Danish versions of two self-administered generic questionnaires: the Functional Assessment of Chronic Illness Therapy-fatigue (FACIT-fatigue) scale [19] and the 12-item Short Form (SF-12) health survey [20, 21], respectively. The proportion of participants who required an RBC transfusion and the proportion who were compliant with treatment (defined as taking ≥ 80% of the intended doses) was also assessed in both treatment groups.

### Safety assessment

The investigators recorded and assessed all adverse events up until the final follow-up visit including clinical assessment of various laboratory parameters measured at each follow-up visit (complete blood count, C-reactive protein [CRP], alanine aminotransferase [ALAT], bilirubin, sodium, potassium, calcium, phosphate, urea, creatinine, and albumin). An adverse event was defined as an event that occurred or increased in severity after the first dose of medication was administered. Investigators assessed all adverse events for relatedness to trial treatment, severity, and seriousness. Adverse events were categorised using the Medical Dictionary for Regulatory Activities (version 20.0). Safety assessments also included the incidence of hypophosphatemia (phosphate < 2 mg/dL), severe hypophosphatemia (phosphate < 1 mg/dL), and discontinuations due to lack of response or intolerance.

In addition, pre-defined obstetric and perinatal outcomes occurring up to seven days postpartum were retrieved from medical records.

### Statistical analysis

Sample size was determined based on the primary hypothesis that IV iron is superior to oral iron for avoiding anaemia. It was assumed that 95% of the IV iron group and 82.5% of the oral iron group would be non-anaemic (Hb ≥ 11.0 g/dL at all follow-up visits). Using a significance level of 5%, and setting the power to 80%, 100 participants in each treatment group were required to detect a difference between IV iron and oral iron.

Statistical analyses were performed using SAS^®^ version 9.4 (SAS Institute, Cary, NC, USA). Statistical methods were prespecified in a statistical analysis plan and the main pre-planned analyses are described below. All statistical tests were two-sided and performed with a significance level of 5%.

Efficacy analyses were conducted on all randomised participants (i.e., the intention-to-treat analysis set). Based on Kaplan–Meier statistics using scheduled follow-up visits, the proportion of women who were non-anaemic was assessed and continuously multiplied at follow-up visits (i.e., the product-limit survival estimates). The cumulative proportion at the last study visit (i.e., 18 weeks after treatment initiation) reflected the primary endpoint (i.e., avoidance of anaemia throughout follow-up) and was compared between the treatment groups as risk difference with 95% confidence intervals (CIs). Women who were anaemic and/or who received prohibited medication during follow-up (iron formulations other than the trial drugs, erythropoiesis-stimulating agents, or RBC transfusion) were set to failures. Women censored at the final, 18-week visit included those who remained non-anaemic, were lost to follow-up, withdrew consent to participate, or lacked a Hb measurement at this visit. Because additional IV iron was only offered to participants with Hb < 11.0 g/dL, (i.e., those who failed to meet the primary endpoint) only the initial treatment allocated at baseline had an impact on the primary analysis. Least squares mean changes (referred to as mean change hereafter) in haematological parameters, fatigue, and QoL from baseline to each follow-up visit were analysed in the intention-to-treat analysis set using a restricted maximum likelihood-based mixed model for repeated measures (MMRM) approach. Observed data were included for all women and, for women without follow-up measurements, the change from baseline was set to 0 at the first follow-up visit. The model included treatment, week, treatment-by-week interaction and stratum as factors, with baseline value and baseline value-by-week interaction as covariates. Compliance data were summarised with descriptive statistics.

Safety analyses were conducted on all women who received treatment (i.e., the safety analysis set). Incidences of adverse events that were related to, or possibly related to, trial treatment, hypophosphatemia, and of discontinuations due to intolerance of, or a lack of response to, trial treatment were compared between the treatment groups using Fisher’s exact test. Biochemical safety parameters were assessed using the same maximum likelihood-based MMRM used for analyses of haematological parameters.

Baseline demographics and clinical characteristics, and obstetric and perinatal outcomes were summarised using descriptive statistics and compared between the treatment groups (post-hoc) using Fisher’s exact test for categorical data and two-sample *t* test or Wilcoxon rank-sum test, as appropriate, for continuous data.

### Ethics

The trial was approved by the relevant authorities (Danish Medicines Agency and the Danish Scientific Ethics Committees) and was registered in the European Clinical Trials Database (EudraCT no.: 2017-000776-29) and on ClinicalTrials.gov (NCT03188445). The trial complied with Good Clinical Practice guidelines. Participation was voluntary and written informed consent was obtained by the trial investigators from all participants, with personal data protected in accordance with the General Data Protection Regulation.

### Funding

The trial was sponsored and fully funded by Pharmacosmos A/S, Holbæk, Denmark. The sponsor was involved in the study design, analysis and interpretation of the data, and approval of the final manuscript.

Further detailed information about trial methods can be obtained in the published protocol article [22].

## Results

**Fig. 1:**
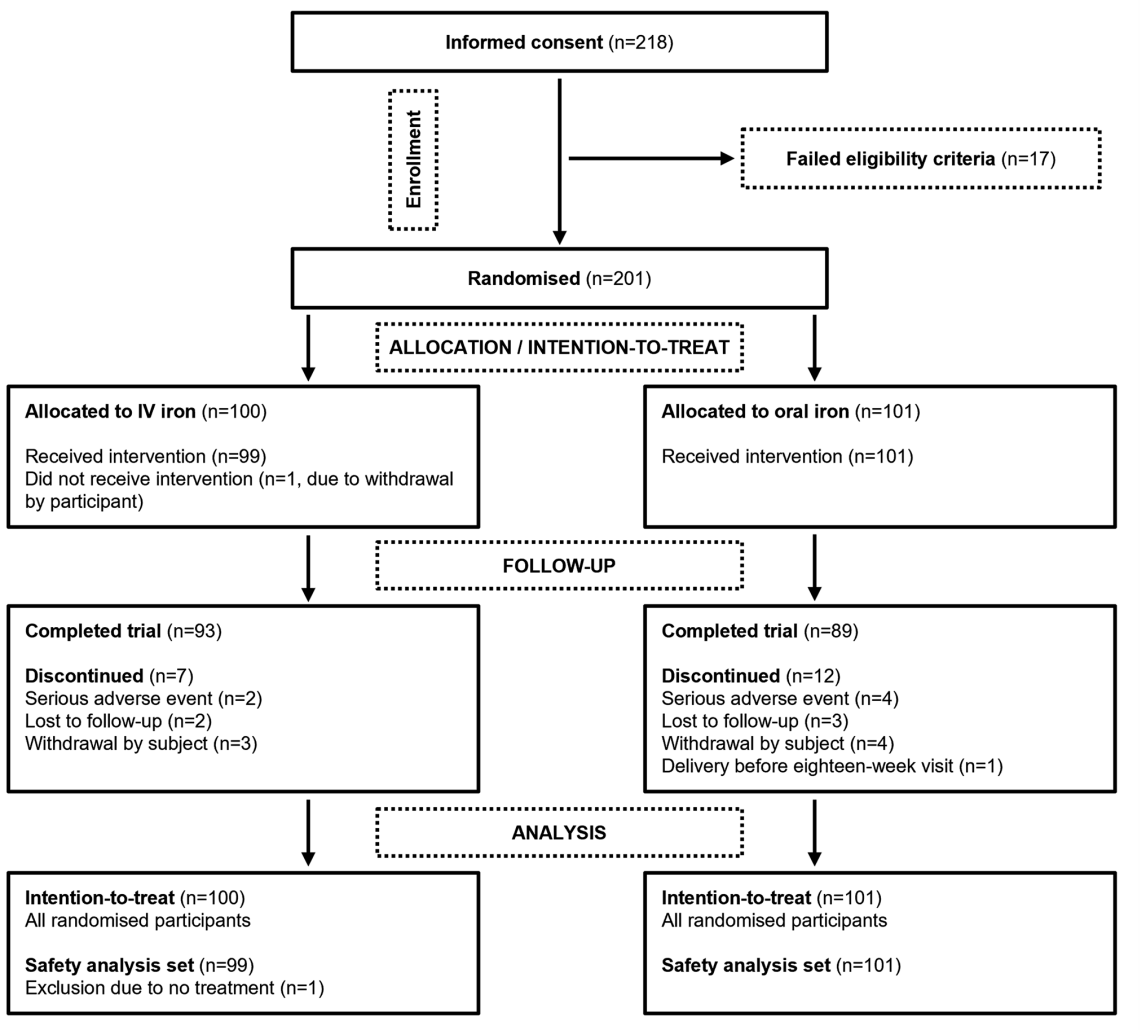
Study flow diagram. All discontinuations due to serious adverse events (*n* = 6) were considered unrelated to trial treatment (preterm birth [*n* = 1] and missed abortion [*n* = 1] in the IV iron group and spontaneous abortion [*n* = 1], preeclampsia [*n* = 1] and preterm birth [*n* = 2] in the oral iron group) *IV* intravenous

From July 2017 to February 2020, a total of 218 women wished to participate and gave informed consent. Eligibility-screening at the baseline visit identified 17 non-eligible women who were excluded before randomisation. Thus, 201 women were randomised to IV iron (*n* = 100) or oral iron (*n* = 101), of whom 93 and 89 women completed the trial, respectively. Based on the last Hb measurement prior to inclusion, 89% of the IV iron group and 88% of the oral iron group were non-anaemic (Hb ≥ 11.0 g/dL). Trial flow, including reasons for discontinuation, is illustrated in Fig. 1. Baseline demographics and clinical characteristics were similar in the two treatment groups (*p* > 0.05) (Table 1).

**Table 1:** Baseline demographics and clinical characteristics

Category	IV iron (*n* = 100)	Oral iron (*n* = 101)
Age (years)		
Mean ± SD	31 ± 5	31 ± 5
Range	21–44	19–42
Race, *n* (%)		
White	88 (88%)	85 (84%)
Asian	10 (10%)	14 (14%)
Black or African American	1 (1%)	1 (1%)
Multiple	1 (1%)	1 (1%)
Smoking,^a^ *n* (%)	13 (13%)	20 (20%)
Dietary preference, *n* (%)		
Vegetarian	7 (7%)	2 (2%)
Vegan		1 (1%)
Neither vegetarian nor vegan	93 (93%)	98 (97%)
Pre-pregnancy BMI (kg/m^2^), *n* (%)	[*n* = 99]	[*n* = 100]
< 18.5	1 (1%)	0 (0%)
18.5–24.9	45 (45%)	47 (47%)
25.0–29.9	35 (35%)	33 (33%)
≥ 30.0	18 (18%)	20 (20%)
Gestational age (days)		
Mean ± SD	132 ± 9	133 ± 10
Range	114–147	109–149
Parity, *n* (%)		
0	40 (40%)	36 (36%)
1	34 (34%)	45 (45%)
2	20 (20%)	18 (18%)
3	6 (6%)	2 (2%)
Time from last delivery (months)		
Mean ± SD	37 ± 24	44 ± 35
Range	6–110	6–156
Stratum, *n* (%)		
Anaemic (Hb < 11 g/dL)	11 (11%)	12 (12%)
Non-anaemic (Hb ≥ 11 g/dL)	89 (89%)	89 (88%)
Haematology, mean ± SD		
Hb (g/dL)	12.0 ± 0.9	11.8 ± 0.9
Ferritin (ng/mL)	21 ± 8 [*n* = 95]	20 ± 7
TSAT (%)	21 ± 10 [*n* = 94]	21 ± 11
PROs, mean ± SD		
FACIT-fatigue score	32.8 ± 9.7	32.0 ± 10.9
SF-12: MCS score	47.7 ± 8.1	47.4 ± 8.2 [*n* = 99]
SF-12: PCS score	47.2 ± 8.6	47.0 ± 9.0 [*n* = 99]

No variables were significantly different between treatment groups using Fisher’s Exact Test for comparison of categorical variables and two-sample *t*-test for comparison of continuous variables (*p* > 0.05)

*BMI* body mass index, *FACIT* functional assessment of chronic illness therapy, *Hb* haemoglobin, *IV* intravenous, *MCS* mental component summary; *PCS* physical component summary, *PRO* patient-reported outcome, *SD* standard deviation, *SF-12* 12-item short-form health survey, *TSAT* transferrin saturation

^a^Within the last 6 months

Of the 100 women allocated to IV iron, 99 received a mean ± SD FDI dose of 982 ± 111 mg (range 150–1000 mg) at the baseline visit. In the oral iron group, the mean ± SD daily elemental iron dose from inclusion to Week 6 was 95 ± 15 mg daily (*n* = 93; 95 ± 15% of the intended dose; range 4–116 mg daily; average total dose 4002 mg), and from Week 6 to Week 18 was 94 ± 9 mg daily (*n* = 72; 94 ± 9% of the intended dose; range 61–121 mg daily; average total dose 7916 mg). Less than 80% of the intended oral iron dose was taken by six participants at the 6-week visit, and four participants at the 18-week visit. Lower dose intake was observed in both treatment groups due to intolerance.

**Fig. 2:**
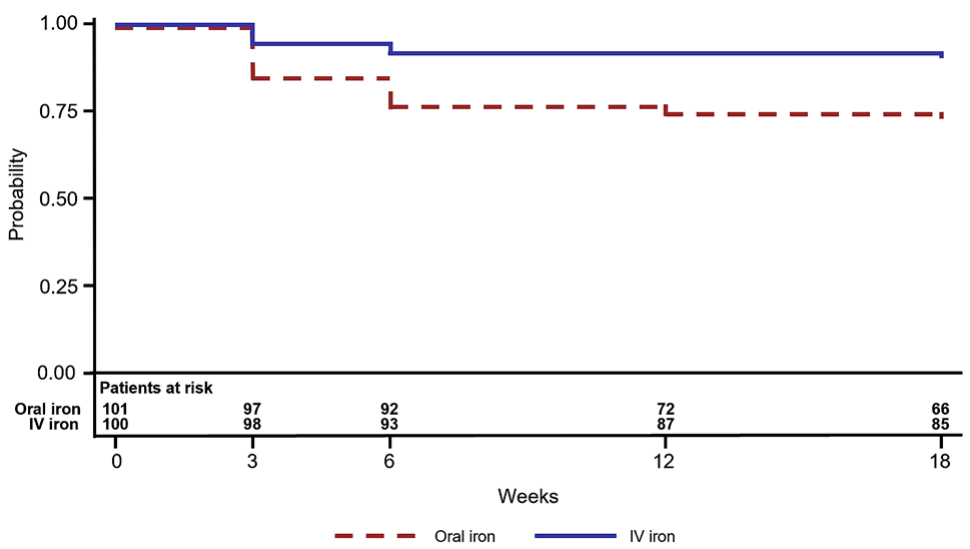
Plots of Kaplan-Meier product limit estimates of avoiding anaemia. Probability of avoiding anaemia post baseline (i.e., Hb ≥ 11.0 g/dL throughout the 18-week follow-up period). *Hb* haemoglobin, *IV* intravenous

The estimated proportion of women with Hb ≥ 11.0 g/dL at all follow-up visits was significantly higher for the IV iron group compared with the oral iron group (91% versus 73%; difference 18% [95% CI 10–25%]; *p* < 0.001) (Fig. 2). Hence, the primary endpoint was met. Kaplan–Meier estimates to support the primary endpoint analysis and Kaplan–Meier plot are summarised in Table S1.

**Fig. 3:**
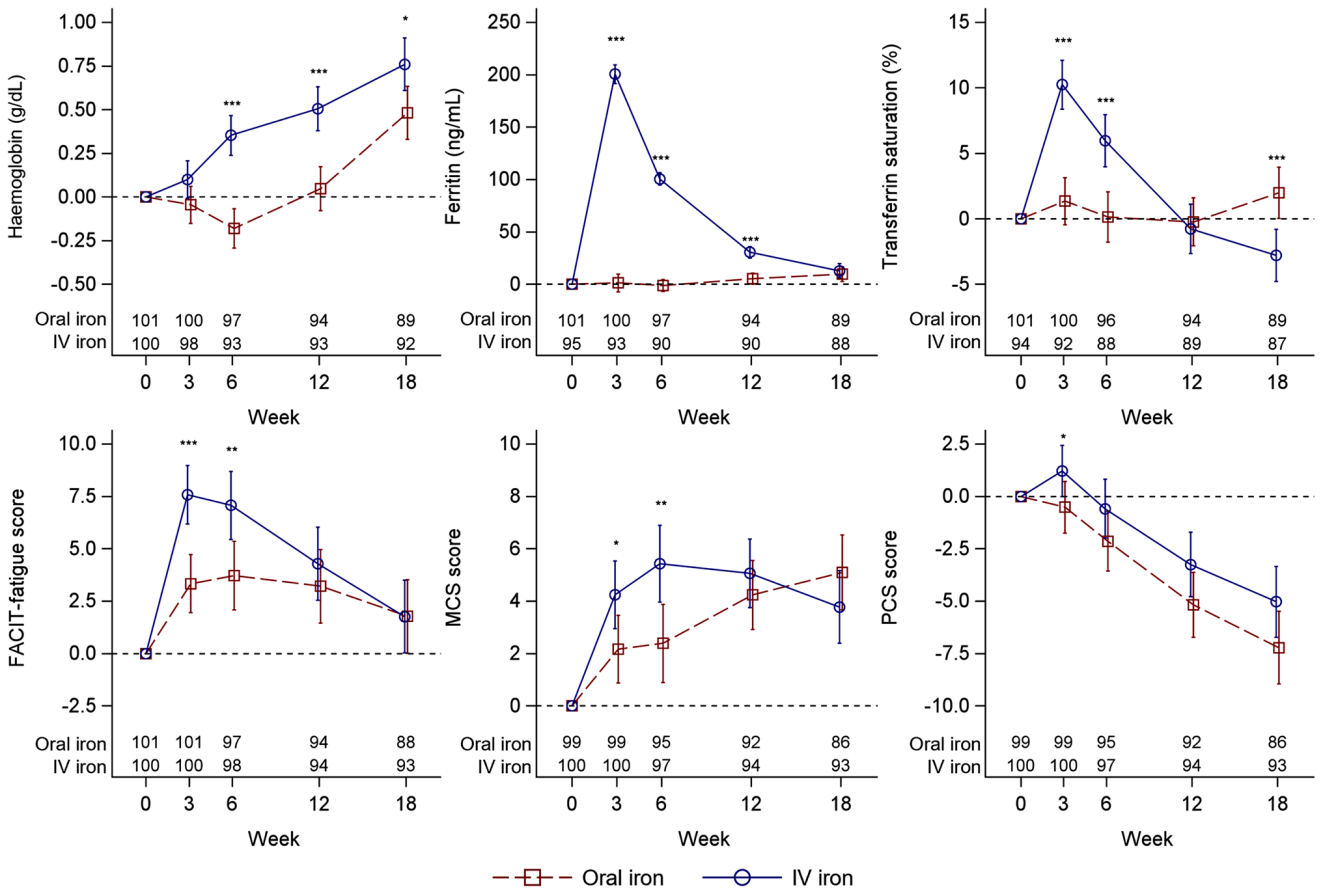
Change in haematological parameters and PROs Mean change values and 95% CI (whiskers) at follow-up timepoints versus baseline with all available samples in the intention-to-treat population included. Changes were assessed and analysed using the restricted maximum likelihood-based MMRM described in the methods section. Number of women contributing with data is given at the bottom. Asterisks indicate significant between-group differences and significance level: *p < 0.05; **p = 0.001–0.01; ***p < 0.001. *CI* confidence interval, *FACIT* functional assessment of chronic illness therapy, *IV* intravenous, *MCS* mental component summary, *PCS* physical component summary, *MMRM* mixed model repeated measures

Mean changes from baseline to follow-up timepoints for Hb, ferritin, TSAT, and PROs are illustrated in Fig. 3 and are summarised in Table S2. The mean Hb increase from baseline was significantly greater in the IV iron group compared with the oral iron group at Weeks 6, 12, and 18 (Fig. 3; Table S2). Following treatment with IV iron, there was a rapid increase in mean ferritin levels from baseline and the change from baseline was significantly greater with IV iron versus oral iron during the first 12 weeks of follow-up (Fig. 3; Table S2). For TSAT, a greater increase was observed in the IV iron group versus the oral iron group within the first 6 weeks of follow-up. However, at Week 18, TSAT decreased in the IV iron group and increased in the oral iron group (Fig. 3; Table S2).

In both treatment groups, FACIT-fatigue scores improved from baseline during follow-up (Fig. 3; Table S2). The mean score difference was statistically significant in Week 3 (4.3 points) and in Week 6 (3.4 points) in favour of the IV iron group, but was not significantly different in Weeks 12 and 18. A similar trend was noted for SF-12 Mental Component Summary (MCS) score: the mean score difference favoured IV iron in Weeks 3 and 6 (Fig. 3; Table S2). In the IV iron group, SF-12 Physical Component Summary (PCS) score increased in the first 3 weeks of follow-up but otherwise decreased in both treatment groups at all follow-up timepoints (Fig. 3; Table S2).

In total, 2 (2%) women in the IV iron group and 20 (20%) women in the oral iron group met the criteria for receiving additional IV iron (i.e., Hb < 11.0 g/dL and ferritin < 30 µg/L at the 6 and/or 12-week visit[s]). The 2 women in the IV iron group and 15 of the 20 women in the oral iron group received the additional IV iron infusion. Of the 15 women in the oral iron group, 10 had taken at least 80% of the oral iron dose intended, 3 had taken less than 80% of the intended dose and, for 2 women, compliance was unknown. Three women in the oral iron group rejected the offer of IV iron, and two remained on oral iron treatment due to safety measures (both women reported a history of allergies during follow-up, which had not been captured at enrolment).

No women received an RBC transfusion while participating in the trial (lowest follow-up Hb measured was 9.5 g/dL).

The proportion of women experiencing adverse events related to, or possibly related to, trial treatment was comparable in the IV iron (43%) and oral iron (47%) groups (*p* = 0.67) (Table S3). No participants withdrew due to intolerance of, or a lack of response to, treatment. Most serious adverse events (*n* = 27/29) were not treatment-related. Two events were considered related to trial treatment (both to IV iron and were classified as hypersensitivity): one was related to treatment administration at baseline (gestational age 20 weeks + 2 days) and the other was related to an additional IV iron administration (gestational age 26 weeks + 5 days). Treatment was immediately terminated when the reactions were recognised. Both reactions were similar, with bronchospasm and flushing arising within the first few minutes of treatment. Furthermore, one of the women experienced lip pruritis and the other facial dysesthesia. Remission of symptoms occurred within a few minutes. Neither of the reactions were accompanied by hypotension or alterations in consciousness. Both women received a single administration of intravenous antihistamine to manage their reaction and one of them also received a single administration of methylprednisolone. Neither of the women received adrenaline and, for both women, all symptoms completely resolved within a few hours. During the infusion reaction connected with an additional IV iron administration, cardiotocography registration quality was poor due to the woman being physically restless. Before and 15 min after the maternal infusion reaction began, the cardiotocography was normal. Both women gave birth at term to healthy children with no maternal or neonatal complications occurring during, or in the first week after, delivery.

Minor between-group mean changes were observed for ALAT, CRP, and albumin, which were not considered clinically relevant. Mean changes in other safety laboratory parameters showed no between-group differences (data not shown). One woman (1.0%) in the IV iron group and three women (3.1%) in the oral iron group had hypophosphatemia (phosphate < 2 mg/dL) at any time after baseline (*p* = 0.62). No cases of severe hypophosphatemia (phosphate < 1 mg/dL) were observed (lowest follow-up phosphate measurement was 1.6 mg/dL). Laboratory parameters considered abnormal and related to, or possibly related to, trial treatment are presented in Table S3.

Most obstetric and perinatal outcomes did not differ between treatment groups (Table S4). Hypertensive disorders of pregnancy (i.e., preeclampsia and gestational hypertension) were more common in the oral iron group than the IV iron group (4% versus 14%; odds ratio: 0.3 [95% CI: 0.1–0.9]; *p* = 0.02) as was gestational diabetes (0% versus 7%; odds ratio: 0.0 [95% CI: 0.0–0.7]; *p* = 0.007). The remaining obstetric and perinatal outcomes, including postpartum haemorrhage, preterm delivery and birth weight, were similar in the two groups (Table S4).

## Discussion

### Main findings

The trial results show that IV iron is superior to oral iron in preventing anaemia and improving Hb and ferritin levels in pregnant women with persistent ID. This is crucial, as ID and IDA in pregnancy can increase maternal and infant morbidity [6–10]. Biochemical effectiveness was accompanied by reduced fatigue and improved psychological well-being within the first 6 weeks of follow-up, and improved physical well-being within the first 3 weeks of follow-up. Overall, no unexpected safety issues were observed with IV iron treatment; transient hypersensitivity reactions abated quickly and without adrenaline treatment, and there was no increased risk of hypophosphatemia.

### Strengths and limitations

A strength of our design was the narrow gestational age range for trial visits, which increased the homogeneity of the data by ensuring that participants were at the same physiological stages of pregnancy at the respective visits. Our trial adds to the knowledge of PROs and clinical outcome measures when comparing IV and oral iron treatment in pregnant women, which has not been described in most previous similar trials. An obvious limitation of our trial was the lack of blinding. It is possible that participants had different expectations of the different treatment types, which may have biased PROs, especially. However, effective and feasible blinding would have been challenging, as IV iron is a dark fluid and oral iron frequently leads to dark-coloured stools. Second, the use of generic questionnaires is a limitation. As there are no existing questionnaires validated for pregnant women that evaluate the burden of symptoms possibly related to ID and IDA, we chose generic questionnaires that seemed most appropriate. Another limitation was the lack of power to measure clinical outcomes such as low birth weight and preterm birth. However, a proper power to investigate the effect of treatment on such rare outcomes would have required an unrealistic number of participants to be randomised. Lastly, the diversity of the study population was limited as most women were white.

### Interpretation

Many of our results are in line with previous findings. Studies evaluating various IV iron preparations for the treatment of IDA in pregnant women have shown consistent improvements in maternal haematological parameters and suggest that hypersensitivity reactions are rare [14, 15]. However, a previous trial in which pregnant women with mixed iron status (ferritin ≤ 100 µg/L) were randomised to receive IV iron sucrose (400–600 mg) or daily oral iron found that the two treatments were equally good at preventing anaemia (80% versus 75% responded to treatment; *p* > 0.05) [23]. These findings contrast the results of the present trial, which suggest that IV iron is superior to oral iron in preventing anaemia. In our trial, we restricted inclusion to women with pre-trial ferritin levels < 30 µg/L, thereby targeting a population with either manifest IDA (*n* = 23) or with non-anaemic ID (*n* = 178) who are at high risk of developing IDA later in pregnancy. Furthermore, we administered IV iron as a single high-dose infusion (1000 mg). These differences may explain our divergent findings.

The oral iron group was highly compliant but, even so, 20 (20%) women in the group had IDA at the 6 and/or 12-week visit(s). Outside a trial setting, it is plausible that compliance would have been lower, probably leading to more severe ID and maybe even IDA in a larger number of women than observed in this trial. Although the ferritin increase in the IV iron group was, initially, rapid, ferritin levels before treatment and at Week 18 of follow-up were similar in most women. This probably indicates that some women needed additional iron treatment, which may be appropriate to administer before ferritin returns to pre-treatment levels.

Few previous trials of IV iron in pregnancy have explored PROs [24, 25] and the reporting of clinical outcome measures is also sparse [15, 16, 26]. Similar to the findings in this trial, Breymann et al. [25] found that QoL improved in favour of IV iron therapy (ferric carboxymaltose) versus oral iron (ferrous sulfate), but this was observed close to term instead of a few weeks after treatment. The FACIT-fatigue scale has been validated in mixed populations with IDA [19] and a change of at least 3 points is thought to reflect the minimal important difference [27]. Therefore, in the present trial, the improvement in fatigue observed in the IV iron group, and the significant difference between groups within the first 6 weeks of follow-up, could be deemed clinically relevant. We did not expect, or power the trial, to find any between-group differences in obstetric and perinatal outcomes. The clinical significance of the higher prevalence of hypertensive disorders and gestational diabetes in the oral iron group compared with the IV iron group is unclear and warrants further study.

## Conclusion

In conclusion, the results of this trial indicate that IV iron is an effective and safe alternative to oral iron for the treatment of persistent ID in pregnant women. Biochemical superiority of IV iron over oral iron treatment in pregnancy has been repeatedly demonstrated and, therefore, future studies should focus on the effects of iron treatment on clinical obstetric and perinatal outcomes, which remain sparsely described.

## Supplementary Information

Below is the link to the electronic supplementary material.

Table S1. Kaplan–Meier estimates to support the primary endpoint analysis and Kaplan–Meier plot, Table S2. Mean changes from baseline to follow-up timepoints for Hb, ferritin, TSAT, and PROs, Table S3. Listing of women with adverse events related to, or possibly related to, trial treatment, Table S4. Obstetric and perinatal outcomes (DOCX 45 KB) Supplementary file1 (DOCX 45 KB)

## Data Availability

The study data will not be available for sharing. The published study protocol is available at https://doi.org/10.1186/s13063-020-04637-z.
