# Supplementary material for: Intravenous ferric derisomaltose versus oral iron for persistent iron deficient pregnant women: a randomised controlled trial
Source: Arch Gynecol Obstet. 2022 Sep 15;308(4):1165–73. doi: 10.1007/s00404-022-06768-x (PMC10435604; doi:10.1007/s00404-022-06768-x)
Supplement: Supplementary file 1 — Table S1. Kaplan–Meier estimates to support the primary endpoint analysis and Kaplan–Meier plot, Table S2. Mean changes from baseline to follow-up timepoints for Hb, ferritin, TSAT, and PROs, Table S3. Listing of women with adverse events related to, or possibly related to, trial treatment, Table S4. Obstetric and perinatal outcomes (DOCX 45 KB) Supplementary file1 (DOCX 45 KB) [file 404_2022_6768_MOESM1_ESM.docx]

**Full article title:** Intravenous ferric derisomaltose versus oral iron for iron deficient pregnant women: a randomised controlled trial

**Short article title:** Intravenous versus oral iron for iron deficient pregnant women

**Journal name:** Archives of Gynecology and Obstetrics

**Authors:** Rebecka Hansen^1^, MD, PhD, Veronika Markova Sommer^1^, MD, Anja Pinborg^2,3^, MD, DMSc, Lone Krebs^1,3^, MD, DMSc, Lars Lykke Thomsen^4^, MD, DMSc, Torben Moos^5^, MD, DMSc, Charlotte Holm^1^, MD, PhD

From: 1. Department of Obstetrics and Gynaecology, Copenhagen University Hospital Hvidovre, Hvidovre, Denmark; 2. Fertility Department, Copenhagen University Hospital Rigshospitalet, Copenhagen, Denmark; 3. Department of Clinical Medicine, University of Copenhagen, Copenhagen, Denmark; 4. Department of Clinical and Non-clinical Research, Pharmacosmos A/S, Holbaek, Denmark; 5. Neurobiology and Drug Delivery, Department of Health Science and Technology, Aalborg University, Aalborg, Denmark.

**Corresponding author:** Charlotte Holm

**Email:** charlotteholm@dadlnet.dk

**Phone:** +4527200832

**Address:** Kettegaard Allé 30, 2650 Hvidovre, Denmark

**Table S1. Kaplan–Meier estimates to support the primary endpoint analysis and Kaplan–Meier plot**

|  | **IV iron** | **Oral iron** |
| --- | --- | --- |
| **Baseline**  Number at risk  Failures  Censored  Product-limit survival estimate (i.e., % non-failures)  Product-limit survival estimate 95% CI | 100  0  2  100%  100 – 100% | 101  1  3  99%  93 – 100% |
| **Week 3**  Number at risk  Failures  Censored  Product-limit survival estimate (i.e., % non-failures)  Product-limit survival estimate 95% CI | 98  5  0  95%  88 – 98% | 97  14  1  85%  76 – 91% |
| **Week 6**  Number at risk  Failures  Censored  Product-limit survival estimate (i.e., % non-failures)  Product-limit survival estimate 95% CI | 93  3  3  92%  84 – 96% | 82  8  2  77%  67 – 84% |
| **Week 12**  Number at risk  Failures  Censored  Product-limit survival estimate (i.e., % non-failures)  Product-limit survival estimate 95% CI | 87  0  2  92%  84 – 96% | 72  2  4  74%  64 – 82% |
| **Week 18**  Number at risk  Failures  Censored  Product-limit survival estimate (i.e., % non-failures)  Product-limit survival estimate 95% CI | 85  1  84  91%  83 – 95% | 66  1  65  73%  63 – 81% |
| **Cumulative numbers at week 18**  Cumulative number of censored (i.e., non-failures)  Cumulative number of failures  Reason for failure  Hb <11.0 g/dL  Prohibited medication | 91  9  8  1 | 75  26  25  1 |
| **Primary endpoint**  Difference in product-limit survival estimates at Week 18 Risk difference  Risk difference 95% CI | 91% vs. 73%  18%  10 – 25% | |

Data used in the Kaplan–Meier method to assess product-limit survival estimates. At post-baseline visits (i.e., after initiation of treatment), women who presented with anaemia were set to failures at the visit when anaemia occurred. In addition, women who received prohibited medication (other iron formulations than the trial drugs, erythropoiesis-stimulating agents, or red blood cells transfusion) were set to failures at the nearest visit up to its administration, which explains the single failure in the oral iron group at baseline. Women who were lost to follow-up or withdrew consent were censored at the following visits. Women with missing Hb were also censored, but only at the visit(s) when the missing occurred. Censored women at the eighteen-week visit additionally included women at risk who had stayed anaemia-free through all post-baseline visits. Hence, the cumulative number of failures included all randomized women with post-baseline anaemia or receiving prohibited medication, and the cumulative number of censored women included all randomized women who did not have anaemia within eighteen weeks post-baseline or those with unknown Hb at the eighteen-week visit (i.e., withdrawals, women lost to follow-up, and women at risk with missing Hb). The cumulative proportion at the eighteen-week visit reflected the primary outcome (i.e., avoidance of anaemia / Hb ≥11.0 g/dL at post-baseline visits) and was compared between the treatment groups as risk difference with 95% CI (primary endpoint analysis).

CI, confidence interval; Hb, haemoglobin; IV, intravenous

**Table S2. Mean changes from baseline to follow-up timepoints**

|  | **IV iron**  **LS Means** | **Oral iron**  **LS Means** | **ΔLS Means**  **95% CI** | **ΔLS Means *p*-value** |
| --- | --- | --- | --- | --- |
| **Haemoglobin (g/dL)**  Week 3  Week 6  Week 12  Week 18 | 0.1 (n=98)  0.4 (n=93)  0.5 (n=93)  0.8 (n=92) | -0.1 (n=100)  -0.2 (n=97)  0.1 (n=94)  0.5 (n=89) | 0.0 – 0.3 **0.4 – 0.7 0.3 – 0.6 0.1 – 0.5** | 0.06  **<0.001**  **<0.001**  **0.01** |
| **Ferritin (µg/L)**  Week 3  Week 6  Week 12  Week 18 | 201 (n=93)  100 (n=90)  31 (n=90)  13 (n=88) | 1 (n=100)  -1 (n=97)  5 (n=94)  10 (n=89) | **187 – 212 93 – 110 18 – 33** -6 – 13 | **<0.001**  **<0.001**  **<0.001**  0.50 |
| **TSAT (%)**  Week 3  Week 6  Week 12  Week 18 | 10 (n=92)  6 (n=88)  -1 (n=89)  -3 (n=87) | 1 (n=100)  0 (n=96)  0 (n=94)  2 (n=89) | **6 – 11 3 – 9** -3 – 2 **-8 – -2** | **<0.001**  **<0.001**  0.69  **<0.001** |
| **FACIT-fatigue**  Week 3  Week 6  Week 12  Week 18 | 7.6 (n=100)  7.1 (n=98)  4.3 (n=94)  1.8 (n=93) | 3.3 (n=101)  3.7 (n=97)  3.2 (n=94)  1.8 (n=88) | **2.3 – 6.2**  **1.0 – 5.7**  -1.4 – 3.6  -2.5 – 2.5 | **<0.001**  **0.005**  0.39  >0.99 |
| **SF-12: MCS score**  Week 3  Week 6  Week 12  Week 18 | 4.2 (n=100)  5.4 (n=97)  5.1 (n=94)  3.8 (n=93) | 2.2 (n=99)  2.4 (n=95)  4.2 (n=92)  5.1 (n=86) | **0.3 – 3.9**  **1.0 – 5.1**  -1.0 – 2.7  -3.3 – 0.7 | **0.03**  **0.005**  0.38  0.19 |
| **SF-12: PCS score**  Week 3  Week 6  Week 12  Week 18 | 1.2 (n=100)  -0.6 (n=97)  -3.3 (n=94)  -5.0 (n=93) | -0.5 (n=99)  -2.2 (n=95)  -5.2 (n=92)  -7.2 (n=86) | **0.0 – 3.5**  -0.4 – 3.6  -0.3 – 4.1  -0.2 – 4.6 | **0.05**  0.12  0.08  0.08 |

Least-squares mean changes: values from follow-up timepoints versus baseline. Changes were assessed and analysed using the restricted maximum likelihood-based MMRM described in the methods section. Bold font indicates statistical significance.

Hb, haemoglobin; IV, intravenous; TSAT, transferrin saturation; SF-12, 12-item Short Form Health Survey; FACIT-fatigue score, Functional Assessment of Chronic Illness Therapy-fatigue scale score; MCS, Mental Component Summary; PCS, Physical Component Summary; MMRM, Mixed Model Repeated Measures.

**Table S3.** **Women with adverse events related to, or possibly related to, trial treatment**

| **Event type, n (%)** | **IV iron  (n=99)** | **Oral iron (n=101)** | **Oral iron + additional IV iron (n=15)** |
| --- | --- | --- | --- |
| **Women at risk, n (%)** | **99 (100%)** | **101 (100%)** | **15 (100%)** |
| **Any related or possibly related adverse event, n (%)** | **43 (43%)** | **47 (47%)** | **5 (33%)** |
| **Gastrointestinal disorders, n (%)**  Constipation  Nausea  Diarrhoea  Abdominal pain  Abdominal pain, upper  Abdominal pain, lower  Dyspepsia  Faeces discoloured  Flatulence  Haemorrhoids  Lip pruritis  Melaena  Vomiting | **11 (11%)**  6 (6%)  2 (2%)  1 (1%)  1 (1%)  1 (1%)  1 (1%)  1 (1%) ^a^  1 (1%) ^b^ | **30 (30%)**  18 (18%)  5 (5%)  5 (5%)  4 (4%)  3 (3%)  1 (1%)  3 (3%)  1 (1%)  1 (1%)  1 (1%) | **1 (7%)**  1 (7%) |
| **General disorders, n (%)**  Drug ineffective  Fatigue  Malaise  Chest pain  Feeling abnormal  Infusion site extravasation  Injection site hematoma  Peripheral swelling  Pyrexia | **11 (11%)**  2 (2%)  3 (3%)  2 (2%)  1 (1%)  1 (1%)  1 (1%)  1 (1%)  1 (1%)  1 (1%) | **15 (15%)**  15 (15%) |  |
| **Nervous system disorders, n (%)**  Headache  Dizziness  Paraesthesia  Syncope  Dysgeusia | **16 (16%)**  10 (10%)  4 (4%)  2 (2%)  2 (2%)  1 (1%) | **1 (1%)**  1 (1%) | **2 (13%)**  1 (7%)  1 (7%) |
| **Skin and subcutaneous tissue disorders, n (%)**  Rash  Pruritis  Eczema  Rash pruritic  Skin discoloration | **6 (6%)**  3 (3%)  2 (2%)  1 (1%)  1 (1%) | **1 (1%)**  1 (1%) | **1 (7%)**  1 (7%) |
| **Investigations, n (%)**  Alanine aminotransferase increased  C-reactive protein increased | **5 (5%)**  4 (4%)  1 (1%) | **1 (1%)**  1 (1%) |  |
| **Metabolism and nutrition disorders, n (%)**  Hypercalcemia  Hypocalcaemia  Hypophosphatemia  Decreased appetite | **2 (2%)**  1 (1%)  1 (1%) | **2 (2%)**  1 (1%)  1 (1%) | **1 (7%)**  1 (7%) |
| **Musculoskeletal and connective tissue disorders, n (%)**  Myalgia  Arthralgia  Musculoskeletal pain | **5 (5%)**  4 (4%)  2 (2%)  1 (1%) |  |  |
| **Vascular disorders, n (%)**  Hypotension  Flushing | **2 (2%)**  1 (1%)  1 (1%) ^a^ |  | **2 (13%)**  2 (13%) |
| **Injury, poisoning and procedural complications, n (%)**  Overdose |  | **3 (3%)**  3 (3%) ^c^ |  |
| **Respiratory, thoracic and mediastinal disorders, n (%)**  Pharyngeal oedema  Bronchospasm | **2 (2%)**  1 (1%) ^d^  1 (1%) ^a^ |  |  |
| **Immune system disorders, n (%)**  Hypersensitivity  Drug hypersensitivity | **1 (1%)**  1 (1%) ^e^ |  | **1 (7%)**  1 (7%) ^e^ |
| **Infections and infestations, n (%)**  Influenza | **2 (2%)**  2 (2%) |  |  |
| **Pregnancy, puerperium and perinatal conditions, n (%)**  Complication of pregnancy | **1 (1%)**  1 (1%) ^f^ |  |  |

Adverse events related or possibly related to trial treatment presented as the number (%) of women experiencing each separate event according to treatment group. Events are summarised as Medical Dictionary for Regulatory Activities (version 20.0) system organ classes (bold) and preferred terms. For subjects in the ‘Oral iron + additional IV iron’ group, events assessed as related or possible related to oral iron are reported in the ‘FA’ group, whereas events assessed as related or possible related to additional IV iron are reported in the ‘Oral iron + additional IV iron’ group.
^a^ Symptom during hypersensitivity reaction
^b^ Reported term: Intermittent black stool
^c^ Taken more than 120% of the dose intended at the six- and/or eighteen-week visits
^d^ Reported term: Pressure in throat
^e^ Serious adverse event
^f^ Reported term: Pruritus gravidarum
IV, intravenous

**Table S4.** **Obstetric and perinatal outcomes**

| **Event type** | **IV iron (n=96)** | **Oral iron (n=95)** | **OR or MD and (95% CI)** | ***p*** ^a^ |
| --- | --- | --- | --- | --- |
| **Antepartum bleeding, n (%)** | 2 (2%) | 1 (1%) | 2.0 (0.2 – 22.4) | 1.00 |
| **Thromboembolic event, n (%)** | 0 | 0 |  |  |
| **Hypertensive disorders of pregnancy, n (%)**  Gestational hypertension  Preeclampsia | 4 (4%)  3 (3%)  1 (1%) | 13 (14%)  8 (8%)  5 (5%) | **0.3 (0.1 – 0.9)** | **0.02** |
| **Gestational diabetes mellitus, n (%)** | 0 | 7 (7%) |  | **0.007** |
| **Length of labour, median hours (IQR)**  Latent phase  Active phase | 6.0 (4.0-11.6)  3.2 (1.1-6.2) | 6.2 (3.5-12.3)  2.2 (1.0-4.6) |  | 0.96  0.17 |
| **Oxytocin use, n (%)**  Augmentation  Treatment for postpartum haemorrhage | 45 (47%)  25 (26%)  27 (28%) | 51 (54%)  29 (31%)  29 (31%) | 0.8 (0.4 – 1.3)  0.8 (0.4 – 1.5)  0.9 (0.5 – 1.7) | 0.39  0.52  0.75 |
| **Postpartum bleeding**  Bleeding (mL), median (IQR) Bleeding ≥500 mL, n (%)  Blood transfusion postpartum, n (%) | 350 (250-500) 29 (30%)  0 | 400 (250-563) 33 (35%)  1 (1%) | 0.8 (0.4 – 1.5) | 0.40  0.54  0.50 |
| **Delivery assisted by vacuum extraction, n (%)** | 7 (7%) | 6 (6%) | 1.2 (0.4 – 3.6) | 1.00 |
| **Emergency caesarean, n (%)** | 11 (11%) | 9 (9%) | 1.2 (0.5 – 3.1) | 0.81 |
| **Deaths, n (%)**  Maternal  Stillbirth/neonatal | 0  1 (1%)^b^ | 0  0 |  | 1.00 |
| **GA at delivery, mean±SD weeks** | 40.1±1.4 | 39.9±1.4 | 0.1 (-0.3 – 0.5) | 0.53 |
| **Preterm delivery (GA<37), n (%)** | 2 (2%) | 3 (3%) | 0.7 (0.1 – 4.0) | 0.68 |
| **Birthweight**  mean±SD  <2500 g, n (%)  >4000 g, n (%) | 3540±437  1 (1%)  11 (11%) | 3653±469  1 (1%)  18 (19%) | -113 (-242 – 16)  1.0 (0.1 – 16.1)  0.6 (0.2 – 1.2) | 0.09  1.00  0.16 |
| **Cord pH**  mean±SD  pH < 7.05 | 7.26±0.08  0 | 7.24±0.08  0 | 0.01 (-0.01 – 0.04) | 0.23 |
| **Cord haemoglobin (g/dL)**  mean±SD | 15.8±1.6 | 15.9±1.9 | 0.0 (-0.5 – 0.5) | 0.94 |
| **Apgar**  Apgar <7 at 5 minutes | 1 (1%) | 0 |  | 1.00 |
| **Labour with foetal distress, n (%)** | 8 (8%) | 4 (4%) | 2.1 (0.6 – 7.1) | 0.37 |
| **Postnatal signs of asphyxia^c^, n (%)** | 4 (4%) | 5 (5%) | 0.8 (0.2 – 3.0) | 0.75 |
| **Paediatric assistance required**^d^**, n (%)** | 7 (7%) | 7 (7%) | 1.0 (0.3 – 2.9) | 1.00 |
| **Neonatal infection^e^, n (%)** | 1 (1%) | 2 (2%) | 0.5 (0.0 – 5.5) | 0.62 |
| **Neonatal anaemia, n (%)** | 0 | 0 |  |  |
| **Neonatal blood transfusion, n (%)** | 0 | 0 |  |  |
| **Congenital malformations**^f^**, n (%)** | 2 (2%) | 2 (2%) | 1.0 (0.1 – 7.2) | 1.00 |
| **Hospital admissions**  Mothers admitted, n (%)  Mothers admitted, median days (IQR)  Neonates admitted, n (%)  Neonates admitted, median days (IQR) | 62 (65%)  1 (0-2)  9 (9%)  0 (0-0) | 57 (60%)  1 (0-2)  9 (9%)  0 (0-0) | 1.2 (0.7 – 2.2)  1 (0.4 – 2.6) | 0.55  0.74  1.00  0.96 |

Pregnancy outcomes presented as n (%) for categorical outcome, and continuous data as mean±SD for normal distributions and median (IQR) for non-normal distributed data. Odds ratios and 95% CI for proportions are calculated by logistic regression with treatment as factor (only estimates from converged models presented). Outcome data available for 96 vs. 95 women in the IV vs. oral iron group except for latent phase (73 vs. 68), active phase (75 vs. 77), cord pH (93 vs. 93), cord Hb (83 vs. 86), and Apgar scores at 5 minutes (96 vs. 94).
^a^ Between-group comparisons: *t* test for mean±SD values, Wilcoxon rank sum test for median (IQR), and Fisher’s exact test for percentage values. Bold font indicates statistical significance.
^b^ Stillbirth. Remained unexplained even after a thorough evaluation including autopsy. Assessed as non-related to trial treatment.
^c^ At least one of the following: respiratory distress, aspiration syndrome, cord pH <7.05 and/or Apgar <7 at 5 minutes.
^d^ At least one of the following: Continuous Positive Airway Pressure, ventilation, intubation and/or resuscitation.
^e^ All three cases were sepsis.
^f^ In the IV iron group one newborn with polydactyly and one with ankyloglossia. In the oral iron group one newborn with one unilateral undescended testis and one child with hydrocephalus and cardiac ventricular septal defect.

IV, intravenous; OR, odds ratio; MD, mean difference; CI, confidence interval; GA, gestational age; IQR, inter quartile range; SD, standard deviation.
